# Genomic investigation of atypical malaria cases in Kanel, northern Senegal

**DOI:** 10.1186/s12936-021-03637-x

**Published:** 2021-02-19

**Authors:** Mouhamad Sy, Aida Sadikh Badiane, Awa Bineta Deme, Amy Gaye, Tolla Ndiaye, Fatou Ba Fall, Katherine J. Siddle, Baba Dieye, Yaye Die Ndiaye, Mamadou Alpha Diallo, Khadim Diongue, Mame Cheikh Seck, Ibrahima Mbaye Ndiaye, Moustapha Cissé, Alioune Badara Gueye, Doudou Sène, Yakou Dieye, Tamba Souané, Bronwyn MacInnis, Sarah K. Volkman, Dyann F. Wirth, Daouda Ndiaye

**Affiliations:** 1 Laboratory of Parasitology and Mycology, Aristide le Dantec Hospital, Cheikh Anta Diop University, Dakar, Senegal; 2 Senegal National Malaria Control Programme, Dakar, Senegal; 3 The Broad Institute of MIT and Harvard, Cambridge, MA USA; 4 Malaria Control and Evaluation Partnership in Africa PATH-MACEPA, Dakar, Senegal; 5 Department of Immunology and Infectious Diseases, Harvard T. H. Chan School of Public Health, Boston, MA USA; 6 College of Natural, Behavioral, and Health Sciences, Simmons University, Boston, MA USA

**Keywords:** *Plasmodium falciparum*, Investigation, Genetic surveillance, Serology, Metagenomic sequencing, Infectious disease

## Abstract

**Background:**

The diagnosis of malaria cases in regions where the malaria burden has decreased significantly and prevalence is very low is more challenging, in part because of reduced clinical presumption of malaria. The appearance of a cluster of malaria cases with atypical symptoms in Mbounguiel, a village in northern Senegal where malaria transmission is low, in September 2018 exemplifies this scenario. The collaboration between the National Malaria Control Programme (NMCP) at the Senegal Ministry of Health and the Laboratory of Parasitology and Mycology at Cheikh Anta Diop University worked together to evaluate this cluster of malaria cases using molecular and serological tools.

**Methods:**

Malaria cases were diagnosed primarily by rapid diagnostic test (RDT), and confirmed by photo-induced electron transfer-polymerase chain reaction (PET-PCR). 24 single nucleotide polymorphisms (SNPs) barcoding was used for *Plasmodium falciparum* genotyping. Unbiased metagenomic sequencing and Luminex-based multi-pathogen antibody and antigen profiling were used to assess exposure to other pathogens.

**Results:**

Nine patients, of 15 suspected cases, were evaluated, and all nine samples were found to be positive for *P. falciparum* only. The 24 SNPs molecular barcode showed the predominance of polygenomic infections, with identifiable strains being different from one another. All patients tested positive for the *P. falciparum* antigens. No other pathogenic infection was detected by either the serological panel or metagenomic sequencing.

**Conclusions:**

This work, undertaken locally within Senegal as a collaboration between the NMCP and a research laboratory at University of Cheikh Anta Diop (UCAD) revealed that a cluster of malaria cases were caused by different strains of *P. falciparum*. The public health response in real time demonstrates the value of local molecular and genomics capacity in affected countries for disease control and elimination.

## Background

The clinical malaria cases reported here occurred in the village of Mbounguiel situated in the department of Kanel within the Matam region. The village is under the responsibility of the Tekkinguel health post located 100 km from the village. However, the villagers go for consultation to the Salalatou heath post, situated 35 km from where the investigated cases were recorded. A health hut is available in the village, but was not functional at the time of the cases, but two trained health care workers were posted to the village in accordance with the outbreak surveillance programme for pre-elimination zones. Indeed, in the North of Senegal, as soon as there is 1 case of malaria an investigation is opened. If there is more than 1 case, a malaria outbreak is declared by the NMCP. The investigation takes place in the house of the index case and the five closest concessions within a radius of 100 m, within 24 h of the case being detected for clinical and socio-anthropological investigation, 72 h for parasitological investigation including molecular tools as well as entomological and environmental investigation. It is carried out by an investigative team [1].

In September 2018, several cases of diarrhoea and vomiting occurred in the village of Mbounguiel. The case investigation started with the reporting of four children who had died after presenting with vomiting and diarrhoea. The first case occurred on September 19th and concerned a male child aged 5 that resided in the village of Mbounguiel. He was hospitalized at the health centre and a malaria diagnosis was confirmed using rapid diagnostic test (RDT) and light microscopy with a parasite density of 88,750 trophozoites/ml. The second case of death occurred on September 20th, in a house in the same village of Mbounguiel and concerned a girl of approximately 6 years of age, but the diagnosis was not established. The two other deaths were reported in a neighbouring village of Babenguel, located 7 km from the village of Mbounguiel, also without diagnosis. Thus, of the four deaths recorded in the community, three were without diagnosis, and one had a positive RDT for malaria.

**Fig. 1:**
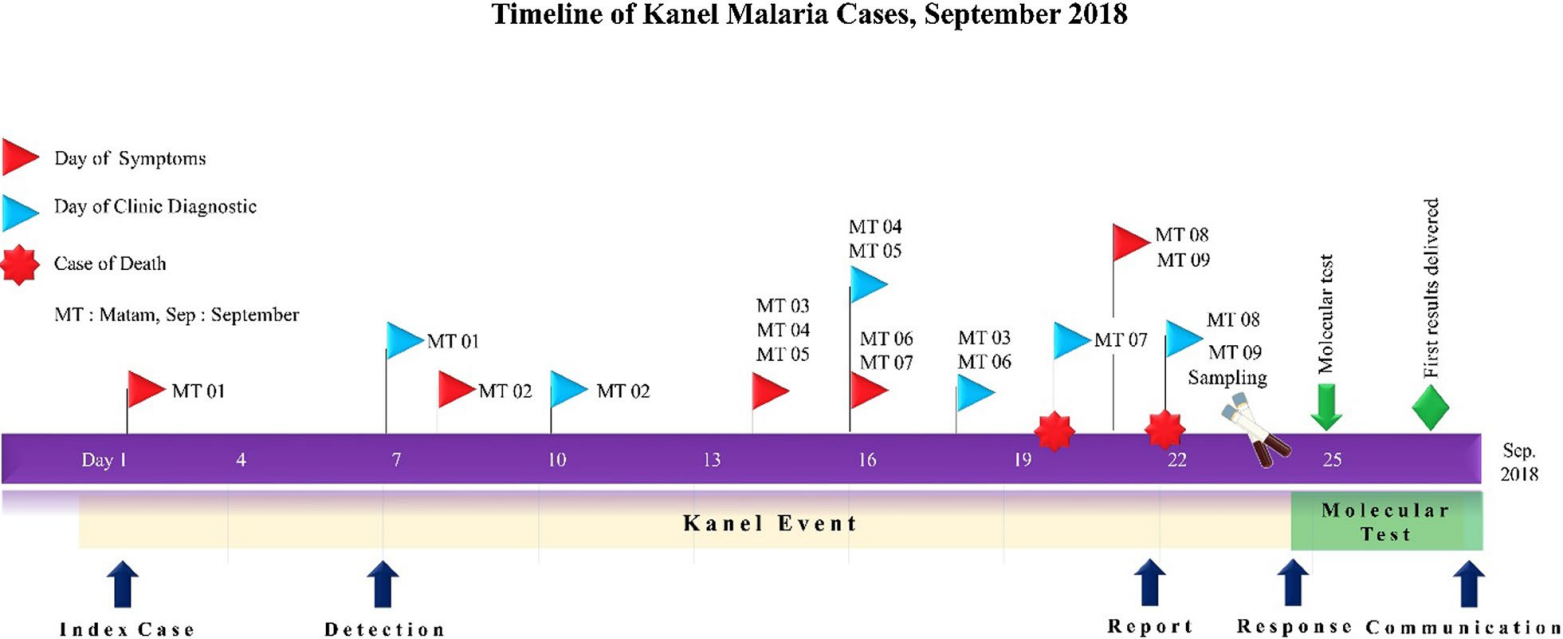
Timeline of Kanel malaria cases, September 2018

Following those four fatalities including one individual with a confirmed malaria case, the health authorities—the Ministry of Health (MoH), the National Malaria Control Programme (NMCP) and the PATH Malaria Control and Elimination Partnership in Africa (MACEPA)—opened a case investigation. A retrospective investigation of the local health clinics of Salalatou identified fifteen people with the same symptoms (diarrhoea, fever, vomiting and jaundice) as the 4 deceased children that were recorded in the same period and all of them were from the same village of Mbounguiel. Of these fifteen people, nine were sampled by the local health team for further investigation. All suspected cases occurred within a 100 to 150 km radius during a period of less than fifteen days, the MoH requested a molecular and serological investigation from the Laboratory of Parasitology and Mycology at Cheikh Anta Diop University to rule out other causes of disease (Fig. 1).

**Fig. 2:**
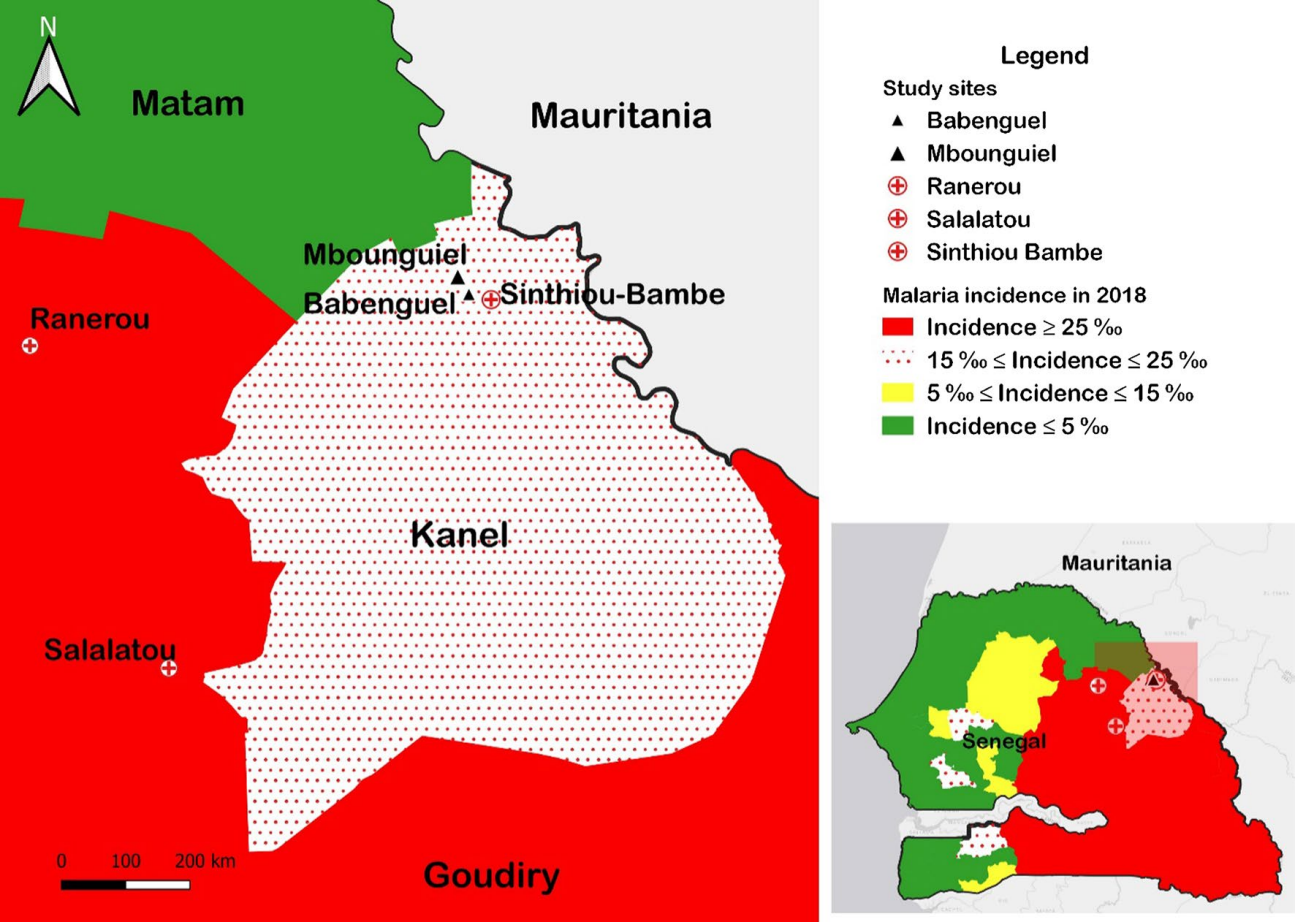
Malaria Incidence Map in 2018 (Source NMCP), showing the location of the region of Matam, the district of Kanel and the village affected by the outbreak and the different health centers. Performed with QGIS 3.4.7 **(**http://www.qgis.osgeo.org)

Northern Senegal, including the regions of Saint-Louis and Matam, is a malaria pre-elimination area with a reported annual incidence below 5/1000 inhabitants. Much of this progress has been made following the implementation of malaria control strategies by the NMCP [2, 3]. The region of Matam is divided into four health districts with different malaria incidences; the district of Matam with 2.5 cases per 1000 inhabitants, the district of Thilogne with 2.3 case per 1000 inhabitants, the district of Ranérou with 68.8 cases per 1000 inhabitants and the district of Kanel with 22.6 cases per 1000 inhabitants in 2018 [2]. The region had a parasite prevalence of 17.2% in 2018 [2] and is bordered to the southwest by the high transmission districts of Koungheul district, Koumpentoum and Tambacounda (with respective incidences of 29.2 cases, 91.9 cases and 225.2 cases per 1000 inhabitants in 2018) (Fig. 2). To sustain malaria control and to reach elimination by 2030 in Senegal there is a need for vigilance in the health system so as to not miss infections, and to prevent sporadic outbreaks and importation into elimination settings, where malaria cases may be more severe due to declining immunity [4].

In such low-transmission areas, surveillance, detection, and early intervention can prevent malaria resurgence and reduce the likelihood of outbreaks [4]. However, light microscopy and/or RDT are of limited use for parasite detection due to the low parasite carriage. Molecular tools like polymerase chain reaction (PCR) can help to overcome this barrier with their lower limits of detection [5, 6]. Different studies have shown the important use of molecular approaches for the detection of malaria and other pathogens, epidemiologic disease comprehension especially during outbreak / hotspot cluster transmission [7, 8], and evaluation of parasite reintroduction by human movement [9, 10]. Molecular tools can also help reveal the causative agent in the context of non-specific symptoms including fever [11].

This study describe the molecular and serological investigation performed in partnership between health ministries, public health authorities and a research laboratory in Senegal to evaluate a cluster of atypical malaria cases in Kanel. Following the establishment of malaria infection by the local medical team using RDT and case investigation in collaboration with PATH-MACEPA, molecular strategies were used to better understand the contribution and characteristics of infectious agents within these samples. For this, different analyses were carried out: (i) photo-induced electron transfer-polymerase chain reaction (PET-PCR) for malaria species detection and typing; (ii) 24 single nucleotide polymorphisms (SNPs) molecular barcode for *Plasmodium falciparum* diversity and possible parasite importation from other locality; (iii) microbead array (MBA) for quantifying antibody response against *P. falciparum* and antibody response against virus causing fever and symptoms like malaria; and, (iv) unbiased metagenomic deep sequencing for virus detection.

## Methods

### Study sites and sample collection

Samples were collected in September 2018 following four deaths that included a case of atypical malaria in the village of Mbounguiel in the department of Kanel, in the region of Matam. The district of Kanel is bordered in the north by the district of Matam which is a malaria elimination site (identified in green on the map) and the high malaria endemic region in the South (in red) (Fig. 2). Malaria transmission in Senegal is highly seasonal with transmission occurring from July to December, and the main causative agent being *P. falciparum* [12]. Recent studies based on serologic data have shown the presence of *P. vivax* in the region of Matam [13], and *Aedes* mosquitoes competent for different dengue serotype transmission have also been isolated in the north [14].

The Ethics Committee of the Ministry of Health in Senegal approved this study. All samples were collected with informed consent per ethical requirements of the National Ethics Committee of Senegal. A venous blood draw (approximately 5 mL in EDTA) was obtained as part of the clinical work up and case investigation from nine suspected malaria cases and was used to perform molecular and serological investigation. Clinical and demographic data was also collected from all suspected cases and included: age, gender, household, treatment received, and outcome. Malaria diagnostic testing (RDT and microscopy) was performed in the field by the regional medical team. Samples were collected by the regional medical team and PATH-MACEPA under the direction and coordination of the NMCP following the NMCP guidance for malaria case instigation [3]. Sample were sent to the Laboratory of Parasitology and Mycology at Aristide Le Dantec Hospital, Dakar.

### Nucleic acid extraction

DNA was extracted from whole blood using the QIAamp® DNA Blood Mini kit (Qiagen ®) according to manufacturer’s instructions. For RNA extraction, an aliquot of the whole blood was first centrifuged to isolate plasma and RNA was extracted from the plasma using the QiAmp® Viral RNA Mini kit (Qiagen).

### PET-PCR

A multiplex photo-induced electron transfer polymerase chain reaction (PET-PCR) assay was used for *P. falciparum* species typing, as previously described [15]. A cycle threshold (CT) value of 40 was used as a cut-off; samples with a CT value less than 40 were considered positive and samples with a CT of 40 or higher were considered negative.

### Molecular barcoding

Samples were pre-amplified using a previously described assay [16]. The molecular barcoding assay was performed on the LightCycler 96 Roche system. All 24 single-nucleotide polymorphisms (SNPs) were amplified as follows; 2.0 μL of Lightscanner Master Mix (BioFire Defense), 2.5 μL of a 1:100 dilution of DNA template, and 0.5 μL of primers and probes. Genomic DNA from cultured *P. falciparum* strains (3D7, Dd2, 7G8, Tm90) was used for assay validation and as genotyping controls for all reaction plates. Molecular barcode assays 10, 11, 13, 21, and 24 were performed optimally under asymmetric forward to reverse primer ratios of 5:1; all other assays required a 1:5 primer asymmetry. Amplification conditions were 95 °C denaturation for 2 min, 50 cycles of 94 °C for 5 s and 66 °C for 30 s, and a pre-melt cycle of 5 s each at 95 °C and 37 °C. Two or more N’s among the 24 SNPs assayed was taken to indicate that more than one *P. falciparum* genome was present [17].

### Serological assays

A multiplexed panel was used to detect the presence of IgG responses to *P. falciparum* antigens and common viruses. All antigens were coupled to magnetic beads (Luminex Corp, TX, USA) for a multiplex bead-based assay (MBA) using MAGPIX technology. For each sample, total immunoglobulin G (IgG) response was measured to four *P. falciparum* antigens, circumsporozoite protein (CSP), merozoite surface protein 1-fragment 19 (MSP1-19), liver stage antigen type 1 (LSA-1), and glutamate-rich protein (GLURP), as well as viruses including Chikungunya virus envelope glycoprotein -1 (CHIKV E1), yellow fever (YFV), dengue virus serotype 2 and 3 ( DENV2, DENV3), Zika virus- like particle (ZIKV-VLP), and West Nile virus (WNV) as described previously [18, 19]. First, a 6 mm circular punch (corresponding to 10μL whole blood) was taken from the centre of each blood spot, eluted in 200μL protein elution buffer B containing: PBS (pH 7.2), 0.05% Tween-20, 0.05% sodium azide and stored overnight at 4 °C until analysis. Next, 50 μL of the bead mixture was added to each well for each plate to assay. The bead mixture contained 6 μL of each bead in 5 mL total Buffer A (0.5% Polyvinyl alcohol (Sigma), 0.8% polyvinylpyrrolidine (Sigma), 0.1% casein (ThermoFisher), 0.5% BSA (Millipore), 0.3% Tween-20, 0.1% sodium azide, and 0.01% *Escherichia coli* extract to prevent non-specific binding). The plate was put on the magnet to allow the fixation of the beads and washed 2 × with 100 μL wash buffer (PBS-T). Then, 50 μL of Reagent Mix (Mix of 10 μL anti-hIgG-BIOT, 8 μL anti-hIgG_4_-BIOT, and 25μL streptavidin-PE to 5 mL Buffer A) was added to each well along with 20 μL of the eluted sample from the Axygen storage plate and 30 μL of Buffer B. In control wells, 50 μL of positive control was added instead. The plate was covered with loose aluminum foil to protect from light, and shaken overnight. The next morning the plate was washed 3 × with PBS and Tween. Finally, 100 μL PBS was added to each well, the plate was shaken for 5 min to resuspend the beads and the assay was read on a MAGPIX machine. Seropositivity was determined as previously described [19] based upon either the distribution of the data (and the antigen), a mixture model, a US non-exposed population, or a serum standard used to determine mean fluorescence intensity (MFI) by subtracting MFI values from blank background beads (MFI-bg). Conventionally, a sample was considered seropositive using a cut-off of rate of antibody expression for each antigen from the US non-exposed population [19].

### Metagenomic sequencing

Extracted RNA was DNAse-treated, cDNA was synthesized, and sequencing libraries were prepared using the Nextera XT kit (Illumina) as previously described [20]. Sequencing libraries were directly constructed from clinical samples without culture or other intervention. Samples were sequenced using Illumina MiSeq with 101 nucleotide paired-end reads at LPM Dakar.

### Sequencing data analysis

Sequencing data were analysed using a publicly-available software viral-ngs v1.25.0 [21], implemented on the DNAnexus cloud-based platform. Individual samples were demultiplexed and reads mapping to the human genome and to other known technical contaminants were removed. KrakenUniq [22], implemented in viral-ngs, was used to identify taxa present in the samples using a database that encompassed the known diversity of all viruses that infect humans, as previously described [21]. A taxon was considered to be present if a greater number of reads from that species were detected in a sample compared to the negative control, after normalizing read counts for the total depth of sequencing, and these reads showed a high k-mer diversity. A de novo genome assembly from raw sequencing reads was further performed, following depletion of human reads, using SPAdes [23], and the resulting contigs were classified using Kaiju. Contigs with a length > 300nt and coverage depth > 10 reads were considered.

## Results

### Characteristics of patients

**Fig. 3:**
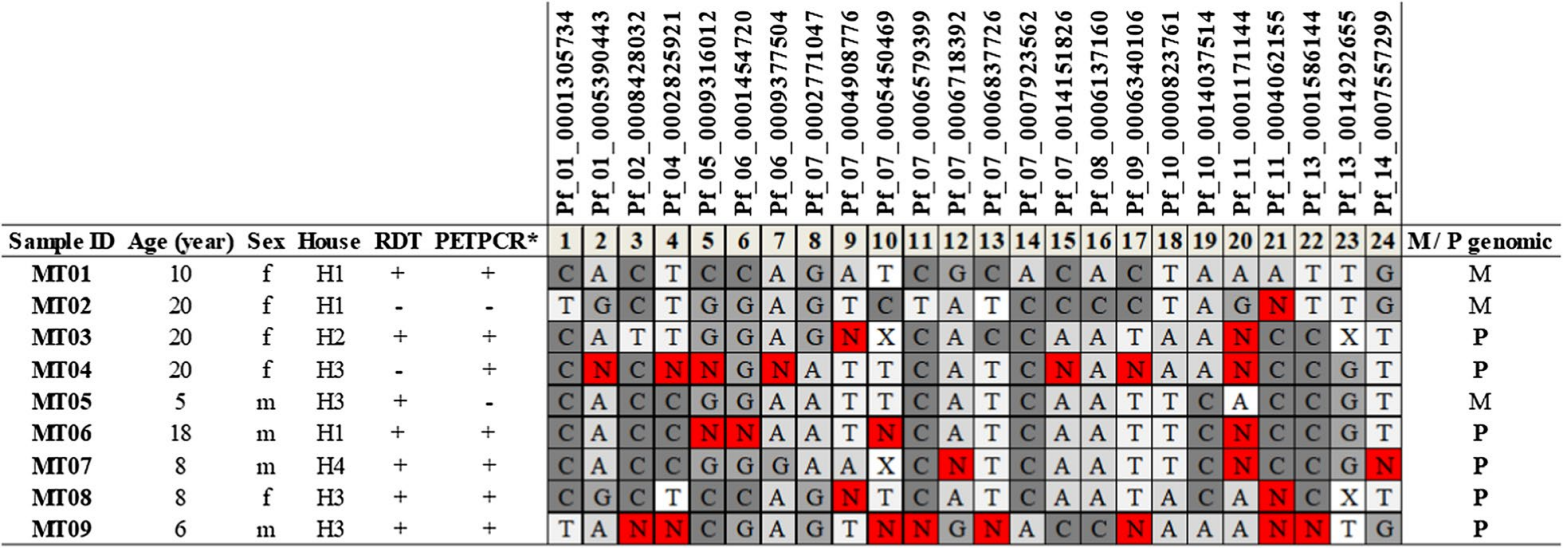
*P. falciparum* rapid diagnostic test (RDT) performed by the local medical team and PET-PCR for malaria species-typing and 24 SNP molecular barcode results. “N” represents a mixed-allele call. “M/P” genomic indicates monogenomic (M) or polygenomic (P) infections, based on the number of Ns; ≥ 2 represents a polygenomic infection. “X” represents a failed assay. H: House, f: female, m: male, M: monogenomic, P: polygenomic

A total of nine samples collected from the suspected cases were sent to the LPM at UCAD for molecular confirmation. Patients were aged between five (5) and twenty-four (24) years (Fig. 3). Cases showed strong clustering at the household level. Three (3) individuals were from H1 (house 1) and four (4) individuals lived in H3 (house 3). All patients presented atypical clinical symptoms for malaria, notably diarrhoea, and other symptoms such as headache, fever, yellowish episodic, vomiting (Fig. 3).

### Molecular investigation

#### Malaria species typing PET-PCR

*Plasmodium falciparum* RDT and PET-PCR gave 7 positive results and 2 negative results. One sample was negative by both RDT and PET-PCR (MT03). Discordant results were observed between RDT and PET-PCR results for MT04 and MT05 (Fig. 3). Of the four different malaria species tested by PET-PCR, all samples were positive for *P. falciparum* only (Fig. 3).

### 24 SNP molecular barcode of *P. falciparum* isolates

All samples were successfully barcoded following pre-amplification. All barcodes were distinct, suggesting no clonal expansion of a specific parasite type among the patients. Polygenomic infections were predominant, and only three (3) patients had a monogenomic infection (MT01, MT02, and MT05) (Fig. 3). Barcodes were compared to a database of barcodes from different regions of Senegal, and none of the nine isolates had been previously identified in Senegal.

### Immunoglobulin G (IgG) response level against *P. falciparum* proteins and other potential viral infections

**Fig. 4:**
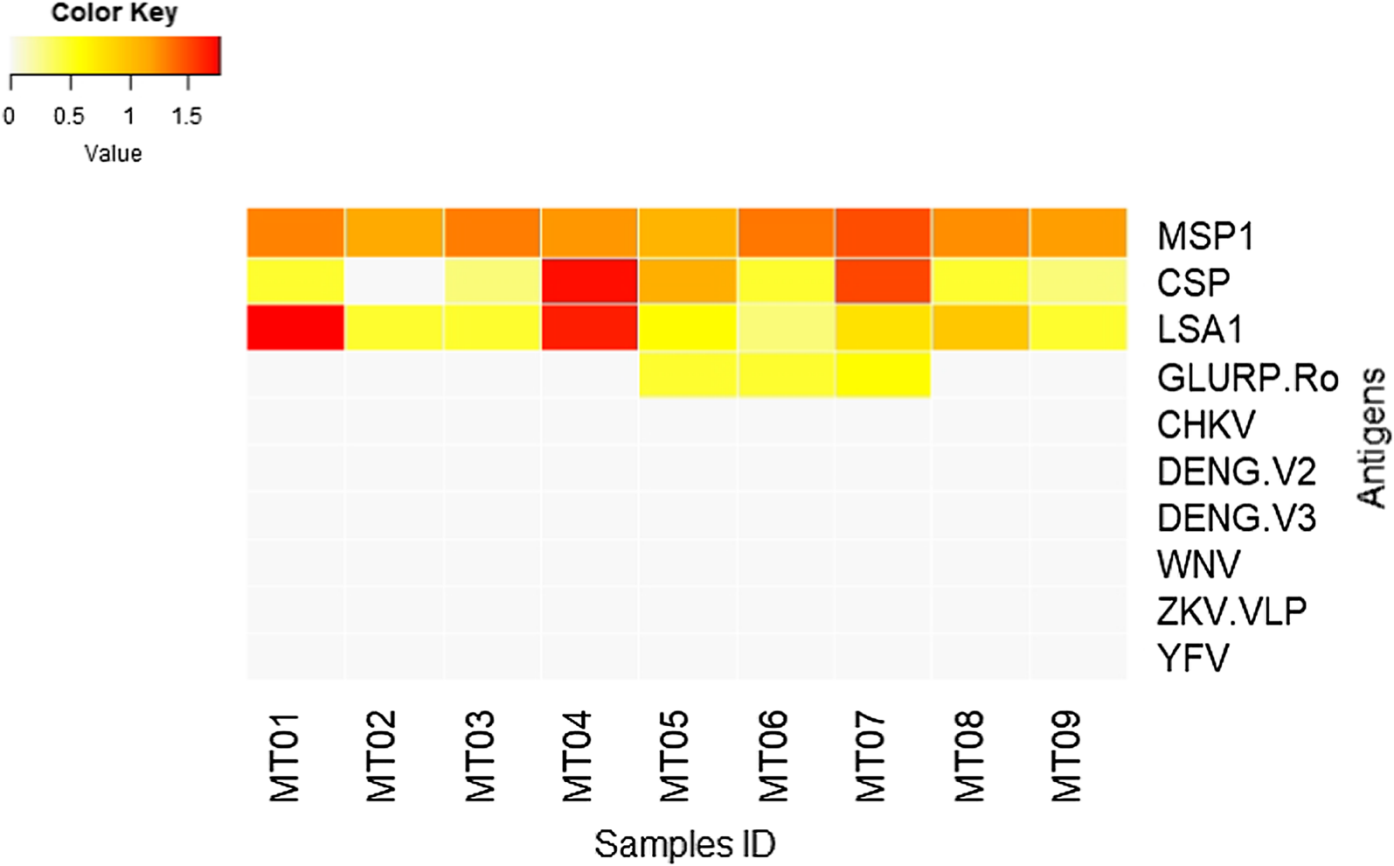
Antibody responses were detected by multiplex bead assay. A strong response to *P*. *falciparum* antigens (MSP1, CSP, LSA1, and GLURP) was observed. All virus antigens tested (CHKV, DENG.V2, DENG.V3, WNV, ZKV.VLP, YFV) were negative

A positive IgG (seropositive) response against *P. falciparum* liver stage (CSP and LSA1) and asexual blood stage (MSP1 and GLURP) antigens was observed among the samples. All patients showed a positive IgG response against MSP, eight patients expressed IgG antibodies against CSP and LSA1, and three patients had IgG antibodies against GLURP. Only two patients (MT05 and MT07) responded positively to all *Plasmodium* antigens tested (Fig. 4). No patient showed an antibody response against any of the viral antigens tested (CHKV, DENV2, DENV3, WNV, ZKV, and YFV).

### Metagenomic deep sequencing

**Fig. 5:**
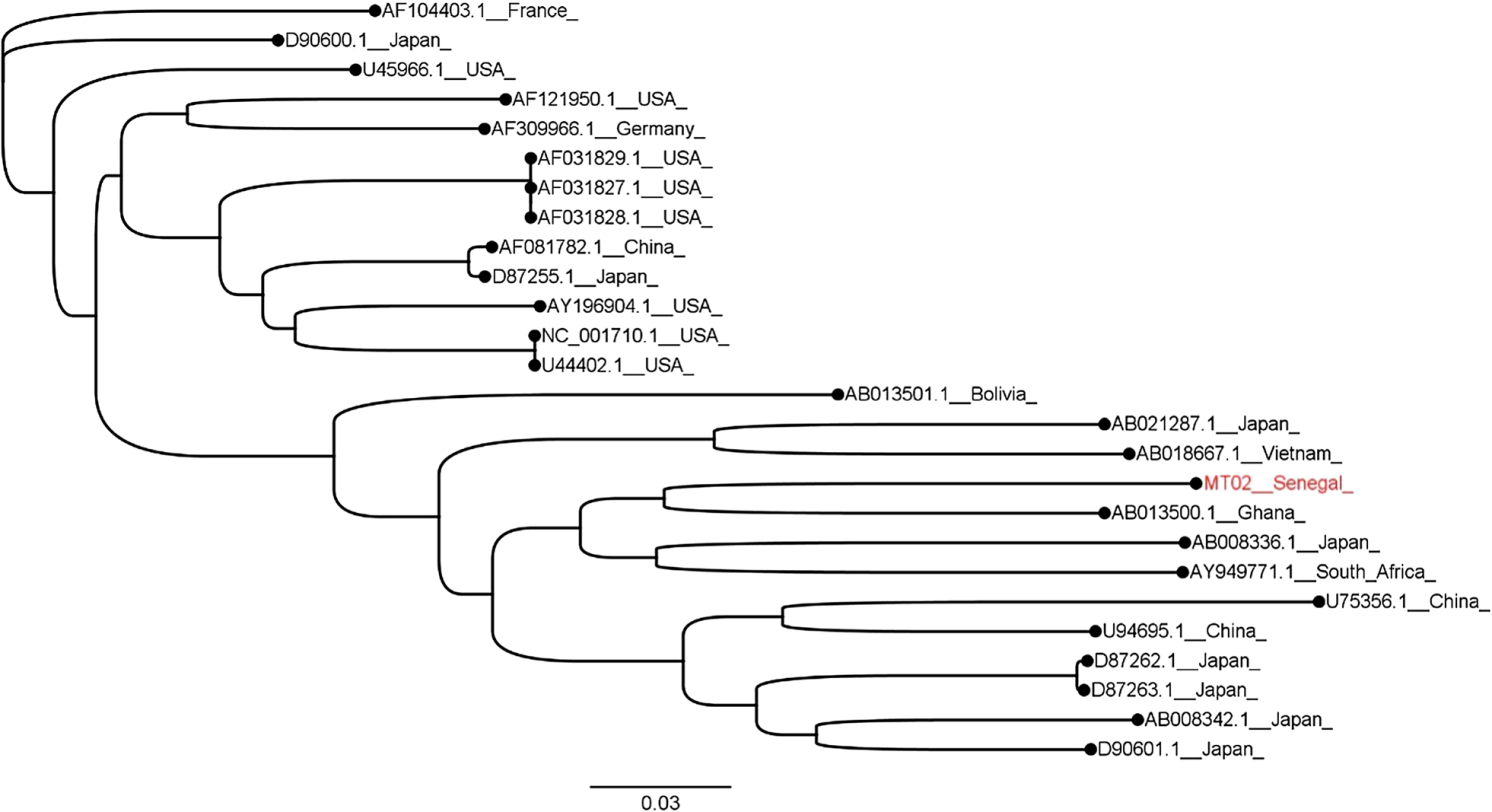
Phylogenetic tree shows the MT02 Pegivirus C (in red text) clustering with an African isolate from Ghana

Metagenomic sequencing was performed to evaluate whether other pathogens were present in the patient samples that could account for the observed symptoms. The metagenomic sequencing did not identify the presence of any other pathogens likely to be related to these cases. However, a non-pathogenic virus identified as a Pegivirus C, which is a common pathogen in healthy individuals [24] was assembled (Fig. 5).

## Discussion

The present study describes the first molecular epidemiological investigation of a presumptive malaria outbreak in the low malaria transmission region of northern Senegal. The findings showed that all cases were associated with malaria infection with *P. falciparum* and no other pathogens were detected. Molecular analysis revealed predominantly polygenomic infections suggesting that the cases were not caused by clonal expansion of an individual isolate. Isolate genotypes did not match barcode genotypes collected elsewhere in Senegal, giving no evidence that these infections were imported.

Using a broad range of different molecular tests enabled the identification of *P. falciparum* as the causal pathogen and removed any suspicion of other possible etiologies, given the unusual symptoms presented by patients. Previous studies have shown that using molecular approaches during outbreaks when patients present with either unusual or non-specific symptoms are crucial in rapidly detecting a pathogen [11]. Similar approaches were used in patients with fever and no specific symptoms to diagnose malaria with RDT and arbovirus using RT-PCR and ELISA [25]. The same study confirmed patients co-infected with malaria and different arbovirus such as Dengue virus, Yellow fever virus, Zika virus, Chikungunya virus, Rift Valley fever virus in the region of Kédougou [25].

In this investigation, a series of tests, both malaria-specific and broad or unbiased assays, showed clearly that this was a cluster of cases of *P. falciparum* infection. The discordant results given by RDT and PET-PCR in two samples could be explained by a difference of sensitivity between the two techniques and also the delay between the malaria diagnosis and treatment and the sampling day (seven days for MT05 and 3 days for MT04). To overcome the low DNA concentration in the samples a preamplification step was performed to study the genetic diversity using molecular barcode genotyping.

The measured IgG antibody response to *P. falciparum* demonstrated past (MSP-1 19) and recent (PfCSP and PfLSA-1) exposure to *P. falciparum* malaria in this population. Previous studies have demonstrated the correlation between circulating *P. falciparum* blood parasites and the presence of anti-MSP-1, anti-CSP and anti-LSA production [26, 27]. Despite the active malaria infection only three patients showed positive IgG responses against *Pf*GLURP antigen. Since two of them were children, five and eight years of age, this could explain a lesser exposure to the parasite, thus less immunity to GLURP which was shown to be correlated with malaria immunity in different studies [28, 29]. A high seroprevalence of *P. falciparum* MSP-1 19, CSP and LSA-1 antibodies was observed in the district of Kanel compared to the other districts in the north suggesting frequent exposure to *P. falciparum* [13]. However, the limited number of samples in this study are insufficient to provide an informative statistical analysis with regard to the level of immunity in this village.

The 24 SNP molecular barcoding revealed unique parasite genetic signatures, showing no clonal expansion. A predominance of polygenomic infections among cases was observed. Similar results were previously observed in the same locality [30]. A large proportion of polygenic infection and high parasite diversity are generally observed in areas of high malaria transmission [10]. In a longitudinal cohort study conducted in Dielmo and Ndiop, molecular barcodes revealed a dramatic change in the parasite population structure; polygenomic infections were observed during the malaria high transmission period and when malaria transmission declined monogenomic infection was observed [31]. The same observation was described in Thiès with monogenomic and clonal infection linked to declining malaria incidence [32]. This unusual trend might suggest that the number of malaria cases during that period is larger than the number that was reported and investigated, and perhaps that local transmission is high. The finding of no shared barcodes between these isolates and the ones isolated from other parts of Senegal and the absence of recent travel history among patients may suggest local, sustained malaria transmission. However, a more extensive investigation of a larger number of samples is needed in this locality to better understand the structure of the parasite population.

The unusual symptoms of diarrhoea and vomiting and the number of reported malaria cases in a short period of time in a low prevalence setting made the medical teams consider either a malaria outbreak or another infectious cause. Given that the North of Senegal is a low prevalence region for malaria, malaria is not the first suspected disease by healthcare workers. The local medical team in collaboration with the NMCP and PATH-MACEPA conducted the investigation in the village, they confirmed the malaria diagnosis, and analysed the factors that could explain this situation. Based on these first results a malaria outbreak was declared in the village and a mass drug administration was conducted in the area.

It emerged from the local investigation that Mbonguiel, where the cases occurred, is far from the official heath post serving the village; the health post in Tikkinguel is around 100 km away. Because of this remoteness and the difficulties of transportation to reach the health post at Tikkinguel,all the cases investigated were diagnosed in the health post of Salalatou located within 35 km and belonging to the health district of Ranerou. It was also noted that during the time of the cases that the health hut in the village itself was not functional due to a lack of community staff. This situation most likely facilitated the spread of the disease in this area. Moreover, in 2018 there were widespread strikes in Senegal beginning in February, resulting in a discontinuity of long-lasting insecticidal nets (LLINs) and the advance strategies of the NMCP could not be conducted because of some logistic problems since the transportation (motorcycle) was non-functional. For better malaria management cases in hard-to-reach areas, health worker can use mobile phones and/or emails to warn the NMCP of an abnormal situation (e.g. cases burden). This will allow a more thorough response from the authorities.

Focal drug administration with artemisinin-based combination therapy (ACT) was carried out in all households (26 houses) within 100–150 m of all positive cases found during the outbreak. A distribution of bed nets was also done to the entire community. Based on the results of the investigation, the ministry of health through the NMCP conducted a distribution of LLINs to the community.

In addition, communication about prevention measures against malaria, in particular the use of the LLIN, was conducted by the local team and it was noticed that in most of the households visited did not have full coverage of all sleeping sites with mosquito nets and those that did exist were not well maintained.

The local investigators made some recommendations to the community and also to the local medical team. The community was also advised to ensure the use of LLINs for the whole family, every night and all year round, to respect the conservation standards of LLINs for good protection of the family. It was recommended that the medical team should make sure that the NMCP recommendations are followed notably the home-based management of malaria (PECADOM) with early implementation of RDTs and ACT in remote villages since 2008 [33], before the start of the rainy season. The community was advised to identify individuals from the community who could be trained as community health care workers. The cases were already diagnosed in the health post and treatment was conducted, however there was the death of four children who are the most vulnerable groups with the pregnant women and only one of those cases was diagnosed at the health center. It is important to establish an earlier diagnosis and treatment of patients with *P. falciparum*-infection as it can reduce malaria complications and stop transmission through rapid clearance of gametocytes [4].

The collaboration between the MoH and the researchers made it possible to further investigae these malaria cases to better understand the relation between the cases.

## Conclusion

The broad range of molecular tools used during this investigation confirmed the presence of *P. falciparum* malaria in all cases and helped to rule out other potential causes within 48 h. As malaria declines in different localities, public health officials will increasingly face situations where molecular techniques will be required to better understand outbreaks and disease origins. This collaboration between the NMCP, the MoH and a local malaria laboratory to confirm malaria outbreak by the use of molecular tools is an example of a strategy that can be replicated more widely to rapidly investigate disease transmission.

## Data Availability

The datasets used during the current study are available from the correspond-ing author on reasonable request.

## Abbreviations

CHKV E 1: Chikungunya virus envelope glycoprotein -1
DENV2: Dengue virus serotype 2
DENV3: Dengue virus serotype 3
WNV: West Nile virus
ZKV VL: Zika virus-like particle
YFV: Yellow fever virus
MSP 1–19: Merozoite surface protein 1-fragment 19
CSP: Circumsporozoite protein
LSA-1: Liver stage antigen type 1
GLURP: Glutamate-rich protein
NGS: Next Generation Sequencing
PCR: Polymerase chain reaction
SNP: Single nucleotide polymorphism
NMCP: National Malaria Control Program
DNA: Deoxyribonucleic acid
cDNA: Complementary Deoxyribonucleic acid
RDT: Rapid diagnostic test
ACT: Artemisinin-based combination therapy
IgG: Immunoglobulin G
MFI: Mean fluorescence intensity
CT: Cycle threshold
MBA: Microbead array
PET: Photo-induced electron transfer
